# A novel hepatocyte ketone production assay to help the selection of nutrients for the ketogenic diet treatment of epilepsy

**DOI:** 10.1038/s41598-024-62723-7

**Published:** 2024-05-24

**Authors:** Hester Meeusen, Alessia Romagnolo, Sophie A. C. Holsink, Thijs J. M. van den Broek, Ardy van Helvoort, Jan A. Gorter, Erwin A. van Vliet, J. Martin Verkuyl, Jose P. Silva, Eleonora Aronica

**Affiliations:** 1 Department of (Neuro)Pathology, Amsterdam UMC, Amsterdam Neuroscience, University of Amsterdam, Amsterdam, The Netherlands; 2 Department of Nutritional Physiology and Functional Nutrients, Medical & Nutrition Science, Danone Nutricia Research, Uppsalalaan 12, 3584CT Utrecht, The Netherlands; 3 Department of Respiratory Medicine, NUTRIM – Research Institute of Nutrition and Translational Research in Metabolism, Maastricht University Medical Center, Maastricht University, Maastricht, The Netherlands; 4 Swammerdam Institute for Life Sciences, Center for Neuroscience, University of Amsterdam, Amsterdam, The Netherlands; 5 Stichting Epilepsie Instellingen Nederland (SEIN), Heemstede, The Netherlands

**Keywords:** In vitro assay, Lipids/oxidation, Liver, Ketogenic diet, β-Hydroxybutyrate, Dietary fat, Nutrition, Omega-3 fatty acids, Preclinical research, Fatty acids, Fat metabolism, Epilepsy

## Abstract

The classic ketogenic diet is an effective treatment option for drug-resistant epilepsy, but its high fat content challenges patient compliance. Optimizing liver ketone production guided by a method comparing substrates for their ketogenic potential may help to reduce the fat content of the diet without loss in ketosis induction. Here, we present a liver cell assay measuring the β-hydroxybutyrate (βHB) yield from fatty acid substrates. Even chain albumin-conjugated fatty acids comprising between 4 and 18 carbon atoms showed a sigmoidal concentration-βHB response curve (CRC) whereas acetate and omega-3 PUFAs produced no CRC. While CRCs were not distinguished by their half-maximal effective concentration (EC50), they differed by maximum response, which related inversely to the carbon chain length and was highest for butyrate. The assay also suitably assessed the βHB yield from fatty acid blends detecting shifts in maximum response from exchanging medium chain fatty acids for long chain fatty acids. The assay further detected a dual role for butyrate and hexanoic acid as ketogenic substrate at high concentration and ketogenic enhancer at low concentration, augmenting the βHB yield from oleic acid and a fatty acid blend. The assay also found propionate to inhibit ketogenesis from oleic acid and a fatty acid blend at low physiological concentration. Although the in vitro assay shows promise as a tool to optimize the ketogenic yield of a fat blend, its predictive value requires human validation.

## Introduction

Whenever energy expenditure is higher than energy intake, for instance during fasting, the body reverts to energy stores. Initially liver and muscle glycogen stores are utilized to provide glucose and eventually fatty acids are metabolized to ketones in the liver. Beta-hydroxybutyrate (βHB), the primary circulating ketone body, has major epigenetic signaling and energetic roles in extrahepatic tissues, such as the brain, heart, and skeletal muscle^1,2^. The switch from glucose to fat utilization and ketone production has been linked to various health benefits, such as extending life and health span, preventing and treating diabetes and cancer^3^. A state of ketosis (> 0.5 mM capillary blood βHB) can be rapidly induced after a short-term fast, or via consuming a well-formulated ketogenic diet (20–50 g/day carbohydrates; 1.2–1.6 g/kg bodyweight protein, ad libitum fat intake to satiety) with or without exogenous ketones^4,5^.

While ketogenic diets (KDs) could benefit various neurological conditions^6^, it is mainly used as a treatment for (drug-resistant) epilepsy, where patients do not achieve sustained seizure-freedom after two adequate trials of anti-seizure drugs. Meta-analyses report seizure freedom in 13% of adults and 33% in infants, and a ≥ 50% seizure reduction in 53% of adults and 59% of infants^7,8^. Multiple anti-seizure actions have been proposed^9^ many of which rely on the presence of ketones^10^.

There have been significant challenges reconciling the anticonvulsant benefits of βHB with dietary adherence in some patients with intractable epilepsy. The “4:1” classic ketogenic diet (cKD) has a 4:1 fat to protein plus carbohydrate weight ratio and consists of 90% calories from fat, 6% from protein and 4% from carbohydrate. While a large proportion of epilepsy centers still use the cKD^11,12^, the exuberant high fat content limits its use in less severe indications. Different versions of the diet, such as the Modified Atkins diet (MAD), the medium chain triglyceride ketogenic diet (MCT-KD) and the low-glycemic index treatment (LGIT), have been developed to lower the fat content. While clinical studies have shown the MCT-KD efficacy is on par with the cKD^13^ and many patients experience significant seizure-reduction using the MAD and LGIT, one more recent study reports sub-optimal seizure suppression with the MAD and LGIT compared to the cKD^14^. Currently, there is insufficient evidence comparing the efficacy of the different KD variants.

Further optimizing the diet in a way where less fat is needed while ketosis and efficacy are maintained could therefore help clinical treatment. The inclusion of Medium Chain Fatty Acids (MCFAs) was an important step towards maximizing the ketogenic potential of the cKD, or the ketone yield in relationship to its fat content. This evidence has been long established and was generated using mostly animal and human pharmacokinetic studies^15–17^. While animal and human studies most faithfully represent dietary metabolic responses and are therefore the best way to compare complete diets or food supplements, the low throughput makes it difficult to compare the contribution to ketogenicity of the individual or blended ingredients. Creating an in vitro assay to screen for the most effective fatty acids and potentially other single nutrients and nutrient combinations could therefore help to optimize their ketogenic potential.

We present here a novel assay that models the production and secretion of ketones from fatty acids by mouse BW7756 liver tumor-derived Hepa1–6 cells (Hepa1–6 cells). The assay allows for the direct comparison of the ketogenic potential of individual fatty acids, and fatty acid blends present in KDs. Liver cells are used in this ketogenic model, as the liver is the primary production and secretion site of ketones for the body. βHB represents the highest circulating fraction of ketone bodies, and therefore serves as the primary readout parameter for the assay. βHB measurements have been previously performed in human derived HepG2 cells^18^ and mouse derived Hepa1-6 cells^19^, but not for profiling ketogenicity of nutrients. An obstacle is the consumption of ketones by tumor-derived hepatocytes. HepG2 cells express the ketolytic enzyme OXCT1^20^, which we also confirmed (data not shown). Their inherent ability to catabolize ketones makes them suboptimal for this assay. In contrast, the expression of OXCT1 and consumption of ketones by Hepa1-6 cells was not reported. Furthermore, we observed a non-zero baseline in ketone production in the absence of a ketogenic substrate in HepG2 cells, indicating the persistence of internal lipid stores even after cellular starvation. The presence of internal lipid stores could interact with the nutrients provided in the assay and interfere with the assay readout. While there are distinct species differences in hepatocyte metabolism and transcriptomics, the main differences described are in increased fatty acid deposition and susceptibility to non-alcoholic fatty liver disease^21,22^. In response to fasting, human and mouse fatty acid metabolism, fatty acid degradation and PPAR signaling seem better conserved^22^.

In this novel assay, we used Hepa1-6 cells to compare the ketogenic potential of most fatty acids present in KDs ranging from short to very long saturated and unsaturated fatty acids. Then, we compared the ketogenic potential of fatty acid blends contained in a classic 4:1 KD, a 6 kcal% MCT-KD (6% MCT-KD) found to protect from traumatic brain injury^23^, and a modified version of the latter comprising 20 kcal% MCT (20% MCT-KD). Furthermore, we assessed ketogenic enhancers produced by the gut microbiome, such as short-chain fatty acids (SCFAs) and certain MCFAs (hexanoic acid, C6). One example is butyric acid (C4), since it drives transcription of FGF21 in liver cells, which in turn upregulates β-oxidation and ketone production in response to fasting or a KD^24,25^. They were tested in combination with ketogenic substrates oleic acid (C18:1) and the 6% MCT-KD fatty acid blend.

## Methods

### In vitro ketogenesis assay

In this ketogenic assay, Hepa1-6 cells (Merck Life Science N.V., Zwijndrecht, the Netherlands) were used. Cells were maintained in 5 mM glucose Dulbecco’s modified Eagle medium (DMEM, Thermo Fisher Scientific, Landsmeer, the Netherlands) to reflect physiological blood glucose levels. The medium was supplemented with 10% fetal bovine serum (FBS, Thermo Fisher Scientific, Landsmeer, the Netherlands) and 1% 10,000 U/ml Penicillin–Streptomycin (Pen-Strep, Thermo Fisher Scientific, Landsmeer, the Netherlands) and cultured at 37 °C in humidified air with 5% CO_2_ in cell culture flasks and passaged every 3–4 days.

**Figure 1:**
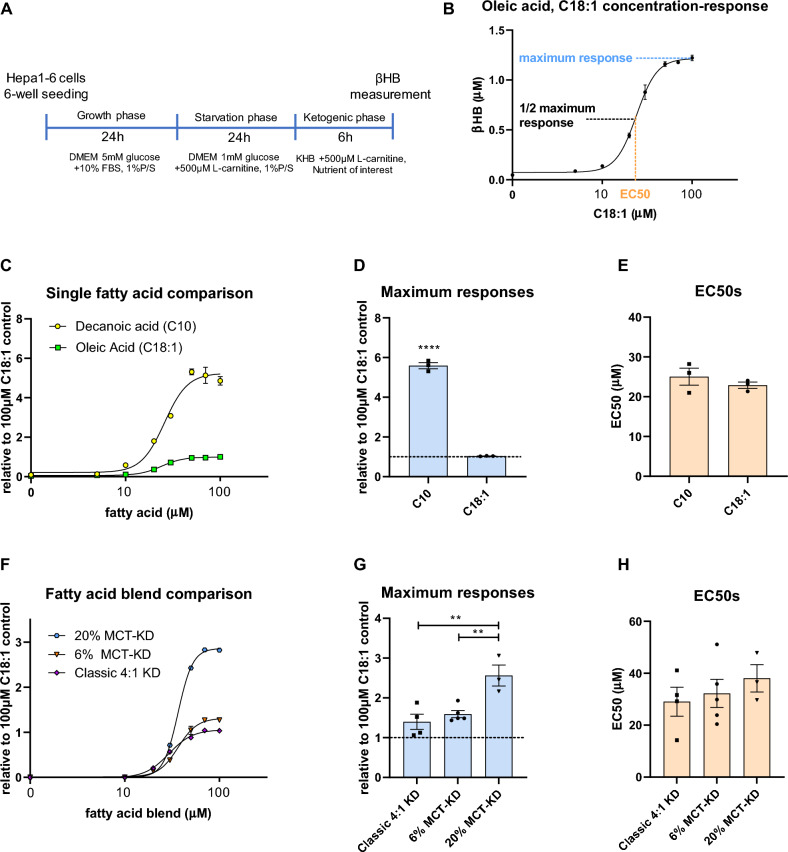
A novel in vitro ketogenic assay. (**A**) Schematic representation of the ketogenic assay. The βHB yield is determined in the cell culture medium at the end of the 6-h ketogenic phase. (**B**) Example of the resulting sigmoidal concentration–response curve (CRC) between C18:1 concentration and βHB yield. The maximum response (blue) refers to the top level yield of βHB after a 6-h incubation, the EC50 (orange) refers to the concentration of fatty acid at which half of the top level βHB yield is achieved. (**C**) Comparison of the CRCs of C10 and C18:1. The single fatty acid comparison experiment consists of an n = 2 per datapoint. The resulting maximum responses (**D**) and EC50s (**E**) of three independent experiments revealed a higher maximum response for C10 compared to C18:1 (****p < 0.0001) in an unpaired *t* test, while it did not reveal differences between EC50s (p = 0.39). (**F**) Comparison of the CRCs of three different fatty acid blends contained in a classic 4:1 KD, and in KDs containing 6 kcal% MCTs (6% MCT-KD) and 20 kcal% MCT (20% MCT-KD) with an n = 2 per datapoint. The maximum response (**G**) of 3- to 5 independent repeat experiments differed significantly and was highest for the 20% MCT-KD fat blend by one-way ANOVA (F (2,9) = 11.5, p = 0.003) and Tukey’s post-hoc test (**p < 0.01) whereas the EC50s (**H**) did not reveal differences in a one-way ANOVA (F (2,9) = 0.5, p = 0.59). In (**C**) and (**F**), the y-axis denotes the βHB yield relative to incubation in 100 µM C18:1. Data are presented as mean ± SEM.

The assay consists of three phases, depicted in Fig. 1A. First, 6-well plates are pre-coated with collagen Type IV (Merck Life Science N.V., Zwijndrecht, the Netherlands) for 1 h at 37 °C and dried before seeding to ensure cell adherence throughout the medium changes of the assay. Hepa1-6 cells were detached using 0.05% trypsin–EDTA (Thermo Fisher Scientific, Landsmeer, the Netherlands) and seeded in a 6-well format at 1.2 × 10^6^ cells per well at 70% confluency, and incubated in 2 ml DMEM 5 mM glucose, 10% FBS, 1% Pen-Strep, and 10% CO_2_ for 24 h. Thereby, the cells adhered without forming islands or clusters and grew into a monolayer. After this growth phase, cells were rinsed with Dulbecco’s phosphate-buffered saline (DPBS, Thermo Fisher Scientific, Landsmeer, the Netherlands) and starved for 24 h in 2 ml serum-free DMEM neither containing glucose nor pyruvate (Thermo Fisher Scientific, Landsmeer, the Netherlands), but supplemented with low 1 mM glucose, 1% Pen-Strep, 500 µM l-carnitine hydrochloride (Merck Life Science N.V., Zwijndrecht, the Netherlands). Thereby, the cells were deprived of glucose, nutrients, hormones, and ketones possibly contained in the serum that would confound the assay readout. This serum and glucose deprivation step forces the cells to consume their glycogen and triglyceride stores and thereby minimizes interference with the assessment of exogenous nutrient effects. Furthermore, glucose restriction activates the β-oxidation and ketogenic pathways. The starvation medium was further supplemented with 500 µM l-carnitine hydrochloride (Merck Life Science N.V., Zwijndrecht, the Netherlands), which is a necessary and rate-limiting cofactor for the translocation of long chain fatty acids (LCFAs) into the mitochondria, where β-oxidation takes place and ketones are produced. The starvation phase lasted for 24 h and was immediately followed by the ketogenic phase. The DMEM was removed, the wells were rinsed with DPBS and switched to 2 ml Krebs Henseleit Buffer (KHB: 555 mM NaCl, 23.5 mM KCl, 10 mM MgSO_4_ and 6 mM Na_2_HPO_4_, 500 µM l-carnitine hyrochloride, pH 7.4, all components from Merck Life Science N.V., Zwijndrecht, the Netherlands). The KHB was further supplemented with a concentration series of a fatty acid or fatty acid blend to obtain a concentration–response relationship. Furthermore, sodium acetate (C2), sodium propionate (C3), sodium butyrate (C4) and hexanoic acid (C6) (all from Merck Life Science N.V., Zwijndrecht, the Netherlands) were added to the buffer at low concentrations (1 µM, 5 µM or 10 µM) in combination with a fixed concentration of ketogenic substrate to assess their concentration-dependent ketogenic enhancement. The ketogenic phase lasted 6 h, after which 2 ml KHB was collected for measuring the amount of βHB secreted into the medium. Since the KHB was devoid of other nutrients, the βHB readout directly related to the test nutrients.

### βHB measurement

To fall within detection range of commercially available kits, the 2 ml KHB samples were concentrated 20 × by evaporating the solvent in a MaxiVac Speed Vacuum Concentrator (LaboGene, Allerød, Denmark) for 24 h at 21 °C, 1000 g and resuspending the pellet in 100 µl assay buffer. The 20 × concentration was corrected for afterwards in the data analysis. The long centrifugation of 24 h did not result in βHB loss. For deproteination, spin columns showed better βHB retrieval compared to metaphosphoric acid precipitation. Samples were deproteinated using 0.5 ml Pierce™ Protein Concentrator microcentrifuge spin columns (Thermo Fisher Scientific, Landsmeer, the Netherlands) at 6000×*g*, 4 °C for 1 h. βHB was measured in an indirect enzymatic and NAD^++^ dependent β-Hydroxybutyrate Fluorometric Assay Kit (Cayman Chemical, Ann Arbor, MI, USA) following the manufacturer’s specifications. This assay specifically measures βHB through its oxidation to acetoacetate by the enzyme 3-hydroxybutyrate dehydrogenase. During this reaction, NAD^++^ is reduced to NADH, which reacts with the fluorometric developer and yields a fluorescent signal that was read at excitation 535 nm and emission at 590 nm using a FlexStation 3 Multi Mode Microplate Reader (Molecular Devices, Reading, UK). The assay does not cross react with other ketone species (acetone, acetoacetate) or structurally similar molecules such as butyric acid that require different enzymes for their oxidation.

### Fatty acid-albumin conjugation

Many fatty acids are barely water-soluble. Therefore, in the circulation they are transported bound to albumin or lipoproteins. These carrier proteins also mediate the cellular uptake of lipids. To test fatty acids in a physiological manner and water-based buffer, they were conjugated to albumin before testing them in the assay. Conjugation of medium, long and very long chain fatty acids to bovine serum albumin (BSA) was performed using an adapted protocol for BSA-palmitate conjugate^26^. Therein, each fatty acid was dissolved in a 150 mM sodium chloride solution (Merck Life Science N.V., Zwijndrecht, the Netherlands) by warming up the solution to 5–8 °C above the fatty acid’s melting point. Additionally, the pH of this solution was increased to 7.4 to deprotonate the carboxyl groups of the fatty acids thereby rendering them more hydrophilic. The fatty acid solution was then added in short bouts to a stirring Ultra Fatty acid-free BSA solution (Merck Life Science N.V., Zwijndrecht, the Netherlands), that never exceeded 40 °C to avoid denaturation of the protein. After stirring for one hour, the BSA conjugated stocks were filtered through a Sartorius Ministart 0.2 μm syringe filter (Merck Life Science N.V., Zwijndrecht, the Netherlands). Due to their inherent difficulty to dissolve in aqueous environments, the final concentrations of all LCFAs were measured afterwards using gas chromatography. C19:0 was used as an internal standard. Fatty acids were converted to methyl esters using methanol and sulphuric acid. After extraction using hexane, the Fatty Acid Methyl Esters were separated and quantified using a gas chromatograph GC-2025 (Shimadzu Benelux B.V., Hertogenbosch, the Netherlands). The ratio between the internal standard and fatty acid methyl esters peaks was used to calculate the concentration of the respective fatty acid stocks used. Under these conditions and by titrating the amounts of added fatty acids, the binding ratio of fatty acid to BSA could be determined, well-controlled and reproduced with minimal inter-experimental variation and could be well compared between different fatty acid-BSA conjugates. The molar binding ratio of fatty acid to BSA was aimed at 4:1 for all conjugates. The SCFAs C2, C3 and C4 were not conjugated to BSA due to their inherent high water-solubility. Aliquots of the saturated fatty acid-BSA conjugate stocks were stored at – 20 °C and unsaturated stocks at − 80 °C. They were diluted to required concentrations in KHB prior to the ketogenic phase. The following fatty acids were used in the assay: C2, C3, C4, C6, octanoic acid (C8), decanoic acid (C10), dodecanoic acid (C12), myristic acid (C14), palmitic acid (C16), oleic acid-albumin from bovine serum (C18:1), linoleic acid (C18:2), α-linolenic acid (C18:3), eicosapentaenoic acid (C20:5) and docosahexaenoic acid (C22:6) (all from Merck Life Science N.V., Zwijndrecht, the Netherlands).

### Concentration–response relationship assessment, internal control and exclusion criteria

To compare the ketogenic potential of fatty acids, first the individual fatty acid concentration response curve (CRC) was determined. This is the relationship between fatty acid concentration and the extracellular βHB production. We tested the most prevalent medium, long and very long even-chain saturated and unsaturated fatty acids contained in KDs and/or supplements, as well as the SCFAs C2 and C4. The tested concentration range was 0, 5, 10, 20, 30, 50, 70, 100 µM. The C4 concentration range was increased to include 200 µM due to an observed right-shift in the curve, relative to the CRC of C18:1. CRCs were generated using Graphpad Prism 8.0.0, by plotting a non-linear fit with variable slope. If the resulting CRC was sigmoidal in shape, the main parameters for comparison were the EC50 and maximum response. An example of a CRC of C18:1 is depicted in Fig. 1B. The EC50 is the fatty acid concentration at which half of the maximum βHB response is reached. It is a characteristic of the potency of a fatty acid. The maximum response is the amount of βHB secreted at the saturated top of the sigmoidal curve. It signifies the maximum capacity of βHB secretion from that specific fatty acid by hepatocytes. For this readout parameter, βHB secretion is expressed as fold-increase over the maximum response of C18:1, which is stably reached at a concentration of 100 µM. C18:1 was chosen as a benchmark as it is the most common mono-unsaturated fatty acid found in natural fat sources, and a main constituent in commercially available KDs. This manner of data expression minimizes interexperimental variation, as the ratios between fatty acid maximum responses are better conserved between experiments than the raw βHB values. Generally, the resulting r^2^ for CRCs is high (> 0.95) if there is no decline in βHB response with increasing fatty acid concentrations above the maximum response. CRCs were excluded with an r^2^ < 0.85. Furthermore, CRCs were excluded when the 100 µM C18:1 control showed a βHB readout < 0.25 µM, as this is below the reliable range of the β-hydroxybutyrate Fluorometric Assay Kit.

We explored whether this assay is well-suited to determine which SCFAs have the potential to enhance the ketogenesis of other fatty acids, and at which concentrations. Low concentrations of SCFAs C2, C3, C4 and the shortest MCFA C6 were tested alone or in combination with 100 µM C18:1 or 100 µM of the 6% MCT-KD fatty acid blend (see results section for composition of the blend), which were supplied as the ketogenic substrate for enhancement. The data is expressed relative to the 100 µM C18:1 control or 6% MCT-KD fatty acid blend in absence of the ketogenic enhancer to facilitate effect-size comparisons between SCFAs.

### Cell viability assessment

To exclude βHB readouts were not confounded by lipotoxic effects of the fatty acids tested in the assay, we performed a cell viability assessment for the single fatty acids used, up to the maximum concentration of fatty acids tested in the assay. The assay was translated to a 96-well format by seeding 4.0 × 10^4^ cells per well. Cells underwent the same assay layout as for ketone production measurements, that is an initial 24-h growth phase followed by a 24-h starvation phase, followed by a 6-h ketogenic phase with 100 µl medium per well. ATP content was measured at the end of the ketogenic phase using the CellTiter-Glo Luminescent Cell Viability Assay (Promega Benelux B.B., Leiden, the Netherlands), following the specifications of the manufacturer. The plate was left to reach room temperature before incubation with CellTiter-Glo reagent to avoid uneven signal development across the plate. Luminescence was measured at 500 nm using a FlexStation 3 Multi Mode Microplate Reader (Molecular Devices, Reading, UK).

### Statistics

For each CRC, the maximum response and EC50 values were averaged and compared to other fatty acids or fatty acid blends using a one-way ANOVA with Tukey’s post-hoc test. With low concentrations of fatty acid provided, the βHB readout generated by the fluorometric assay can show values slightly below 0, these were set to 0 in the analysis. For the saturated fatty acids from C4 up and till C18, the correlation with βHB production was determined using the Spearman’s Rho test and visualized using the exponential one phase decay model, with the plateau constrained to 0 (GraphPad Prism version 9.5.0).

The combined βHB yield of SCFAs and fatty acid substrate (C18:1 or the 6% MCT-KD fatty acid blend) was compared to the βHB yield of the fatty acid substrate alone using a one sample t-test. Additionally, we determined whether the SCFAs acted as ketogenic enhancers, that is whether the βHB yield was more than additive. The sum of the βHB yield from incubating the SCFAs or fatty acid substrate (C18:1 or the 6% MCT-KD fatty acid blend) alone, was compared to the βHB yield of their respective coincubation using a paired t-test.

## Results

### The in vitro ketogenic assay

The assay layout of the ketogenic assay, which uses Hepa1-6 cells, is detailed in Fig. 1A. Exposure of the cells to a concentration range of oleic acid (C18:1) produced a sigmoidal CRC with an EC50 value and maximum response value (Fig. 1B). We assessed the ketogenic response of single fatty acids and fatty acid blends by comparing their EC50 and maximum response parameters to C18:1 as the internal reference run in parallel. Examples of the CRC curves are shown for the MCFA decanoic acid (C10) along with C18:1 (Fig. 1C), with the EC50s and maximum responses of three experiments. C10 showed a 5.6-fold increase in the maximum response compared to C18:1 (p < 0.0001, unpaired *t* test; Fig. 1D), while the EC50s were the same (p = 0.394, unpaired *t* test; Fig. 1E). This data thus shows that the assay can detect differences in the ketogenic potential of single fatty acids, mainly in the maximum response parameter.

Next, we compared three fatty acid blends contained in a classic 4:1 KD, a 6 kcal% MCT-KD (6% MCT-KD) and a 20 kcal% MCT-KD (20% MCT-KD). Table 1 shows the molar composition of the fatty acids contained in these blends. For both MCT-KDs, the molar content of LCFAs was exchanged for MCFAs (C6-C12) to 7.2 mol% and 26.2 mol%, respectively, compared to 0 mol% in the classic 4:1 KD. Both the 6% and 20% MCT-KD blends are enriched with coconut oil.

**Table 1:** Molar composition of three different fatty acid blends contained in a classic 4:1 KD, and in KDs containing 6 kcal% MCTs (6% MCT-KD) and 20 kcal% MCT (20% MCT-KD).

	C4	C6	C8	C10	C12	C14	C16	C18	C18:1	C18:2	C18:3	C20:5	C22:6
Classic 4:1 KD	0	0	0	0	0	0.7	32.6	4.5	31.6	27.5	2.9	0	0.2
6% MCT-KD	0	0.1	1.7	1.4	4.0	2.5	34.7	4.4	37.1	9.7	1.0	0.6	2.8
20% MCT-KD	0	0.2	6.3	5.0	14.7	2.0	27.6	3.5	29.5	7.7	0.8	0.5	2.2

The table shows the molar percentage (mol%) of each fatty acid contained in each blend with a total of 100%

Figure 1 shows a representative example of the CRCs (Fig. 1F) with mean maximum responses (Fig. 1G) and mean EC50s (Fig. 1H) of N = 3–5 independent experiments per fatty acid blend. As expected, the 20% MCT-KD yielded a higher maximum response than the 6% MCT-KD and 4:1 classic KD (F (2, 9) = 11.45, p = 0.0034, one-way ANOVA; p = 0.0037 vs 4:1 KD, p = 0.0085 vs 6% MCT-KD, Tukey’s posthoc test) while the maximum response of the 6% MCT-KD and the 4:1 classic KD were similar (Fig. 1G). The EC50s did not differ between fat blends (F (2, 9) = 0.562, p = 0.589, one-way ANOVA; Fig. 1H). Therefore, the assay can characterize differences in ketone production from fatty acid blends.

### Assessment of single fatty acids

**Figure 2:**
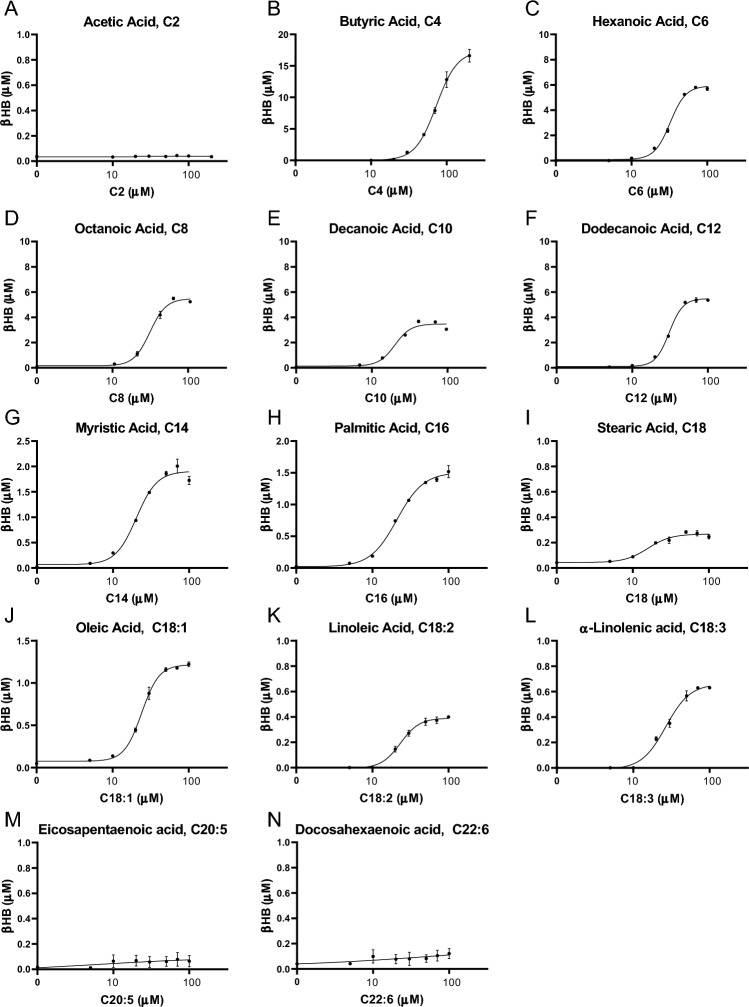
Single fatty acid CRCs. (**A**–**N**) Examples of single fatty acid CRCs tested in the ketogenic assay, with an n = 2 per datapoint. All single fatty acids except C2, C20:5 and C22:6 showed a sigmoidal CRC. To improve visibility, the scale of the y-axis denoting the βHB concentration was adjusted.

**Figure 3:**
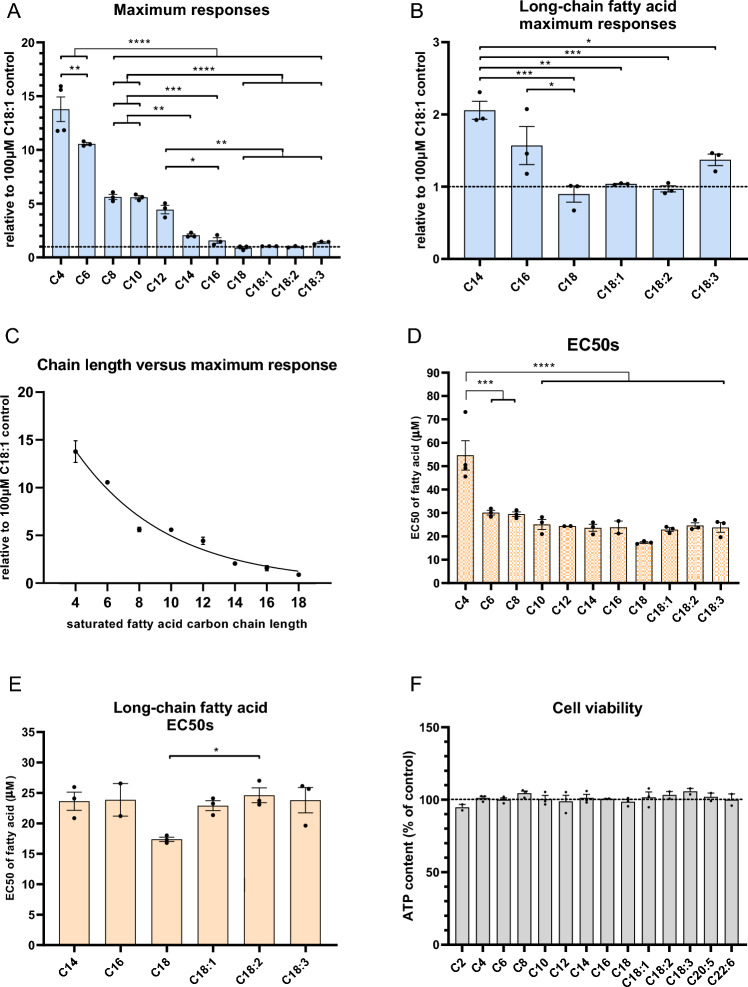
Ketogenic differences between single fatty acids. (**A**) The resulting maximum responses of the single fatty acid CRCs of at least three independent repeats are expressed relative to the maximum response achieved with 100 µM C18:1, which was run in parallel with each assessment, and differ by one-way ANOVA (F(10,23) = 83.31, p < 0.0001). All fatty acids were compared to each other by Tukey’s post-hoc test with significance levels indicated in the graphs (*p < 0.05, **p < 0.01, ***p < 0.001, ****p < 0.0001). (**B**) The maximum responses of LCFAs were analyzed separately by one-way ANOVA (F(5,12) = 11.16, p = 0.0004) and compared using a Tukey’s post-hoc test, showing highest βHB production for C14 compared to all C18 variants (*p < 0.05, **p < 0.01, ***p < 0.001) and increased βHB production for C16 compared to C18 (*p < 0.05). (**C**) The maximum βHB yield (relative to that of 100 µM C18:1) of even-chain saturated fatty acids from C4 until C18 was inversely correlated to the carbon chain length (Spearman’s correlation coefficient *r* = 1, p = 0), and curve fitted with the equation: Y = 27.6 × exp(− 0.172 × X) (**D**) The EC50s of the single fatty acid CRCs of at least three independent repeats showed differences by one-way ANOVA (F(10,21) = 1, p < 0.0001). Tukey’s post-hoc test comparing all fatty acids showed the highest EC50 for butyrate (C4; ***p < 0.001, ****p < 0.0001). (**E**) The EC50s of LCFAs were analyzed separately by one-way ANOVA (F(5, 11) = 3.421, p = 0.0415), and compared by Tukey’s post-hoc test, and was lowest for C18 compared to C18:2 (*p < 0.05). (**F**) Cell viability assessment at the end of the ketone production assay, following exposure to single fatty acids at their highest concentration (200 μM) used in the assay, with 2 to 3 independent repeats per fatty acid. Cell viability was determined by measuring the total ATP content and was similar between fatty acids (one-way ANOVA, F(13,22) = 0.90, p = 0.56). Data are presented as mean ± SEM.

We measured the βHB yield from saturated short (C2–C4), medium (C6–C12) and LCFAs (C14–C18), unsaturated LCFAs (C18:1, C18:2. C18:3) and unsaturated VLCFAs (C20:5, C22:6) in the assay. A concentration range from 1 µM up to 100 µM, and 200 µM for C2 and C4 was covered. Since liver cells were incubated with one specific fatty acid and l-carnitine in the absence of other nutrients in Krebs Henseleit buffer (Fig. 1A), the βHB yield in the medium at the end of the 6-h incubation was assumed to reflect utilization of this fatty acid as the ketogenic substrate. Figure 2 shows representative examples of the CRCs. Most fatty acids showed a sigmoid CRC, indicating a dose–response relationship. C2, C20:5 and C22:6 barely yielded βHB, indicating poor use as ketogenic substrates. Figure 3A shows the maximum response of each fatty acid. To account for inter-experimental variation, the maximum responses were normalized to the ketogenic response to 100 µM C18:1, which was assessed as an internal reference with each CRC assessment. The maximum responses were significantly different between fatty acids (F(10,23) = 83.31, p < 0.0001, one-way ANOVA). C4 showed the highest maximum response with a 13.8-fold greater βHB yield over C18:1 (p = 0.0031 vs C6; p < 0.0001 vs all other fatty acids; Tukey post-hoc test). C6 displayed the second highest maximum response with a 10.6-fold increase in βHB yield over C18:1 (p < 0.0001 vs all longer fatty acids; Tukey post-hoc test). C8 and C10 showed the third highest maximum response, with similar performance (p > 0.99; Tukey post-hoc test). Both increased the βHB yield 5.6-fold over C18:1 and performed better than C14 (p = 0.02), C16 (p = 0.0005), C18 (p < 0.0001), C18:1 (p < 0.0001), C18:2 (p < 0.0001), and C18:3 (p = 0.0002) as determined by Tukey post-hoc test. C12 increased the maximum response 4.4-fold over C18:1 (p < 0.0001). C12 tended to be slightly less performant than C8 and C10 (p = 0.8 by Tukey post-hoc test; p = 0.062 vs C8 by *t* test, p = 0.052 vs C10 by *t* test) and more performant than C14 (p = 0.08 by Tukey post-hoc test; p = 0.0043 by *t* test), while eliciting a greater response than C16 (p = 0.019), C18 (p = 0.002), C18:1 (p = 0.0035), C18:2 (p = 0.0028), and C18:3 (p = 0.01).

LCFAs were also compared between them only (Fig. 3B). This revealed a difference in maximum response (F(5,12) = 11.16, p = 0.0004 by one-way ANOVA). Of all LCFAs, C14 displayed the highest maximal response compared to C18:1 with a 2.1-fold higher βHB yield (p = 0.0017, Tukey’s post-hoc test; Fig. 3B). C16 displayed the second highest maximal response of all LCFAs with a 1.6-fold higher βHB yield than C18:1, although it was only significantly elevated compared to C18 (p = 0.035, Tukey’s post-hoc test; Fig. 3B). The βHB yields from C18, C18:1 C18:2, C18:3, were similar.

When the maximum response was related to the carbon chain length of saturated fatty acids comprising between 4 and 18 carbon atoms, an inverse correlation was found (Spearman rank correlation coefficient *ρ* = 1, p = 0; Fig. 3C) with the following equation: Y = 27.6 × exp(− 0.172 × X), whereby Y is the ketone yield relative to that of 100 µM C18:1, and X represents the carbon chain length (2, 4, 6, 8, 10, 12, 14, 16 or 18). This is in line with the notion that MCFAs are more ketogenic than equimolar amounts of LCFAs. Meanwhile the saturation of fatty acids containing 18 carbon atoms did not correlate with the maximum response (Spearman rank correlation coefficient *ρ* = 0.8, p = 0.2).

Although the EC50s of the CRCs differed (F (10, 21) = 12.6, p < 0.0001, one-way ANOVA), this difference was only due to C4 (p = 0.0002 vs C6, p = 0.0001 vs C8, p < 0.0001 vs all other fatty acids, Tukey post-hoc test; Fig. 3D) while 2-group comparisons excluding C4 were not significant (p > 0.16, Tukey post-hoc test). The EC50 of C4 averaged 55 μM while the EC50s of all other fatty acids ranged between 17 μM and 30 μM (Fig. 3D,E), with a mean of 27 μM. In addition, the EC50s of LCFAs were analyzed separately (Fig. 3E) and were significantly different by one-way ANOVA (F(5, 11) = 3.421, p = 0.0415). C18 had a lower EC50 compared to C18:2 (*p < 0.05, Tukey’s post-hoc test). Hence, the assay detected differences between fatty acids mainly in the maximum response parameter.

Since fatty acids could be cytotoxic and skew the ketogenic profiling, the ATP content as a proxy for the number of alive liver cells was measured at the end of the 6-h ketogenic phase of the assay. The ATP content was similar between fatty acid exposures (F (13, 22) = 0.903, p = 0.563, one-way ANOVA; Fig. 3F). Hence, differences in maximal responses were not confounded by cytotoxicity.

### Enhancement and restriction of ketone yield

**Figure 4:**
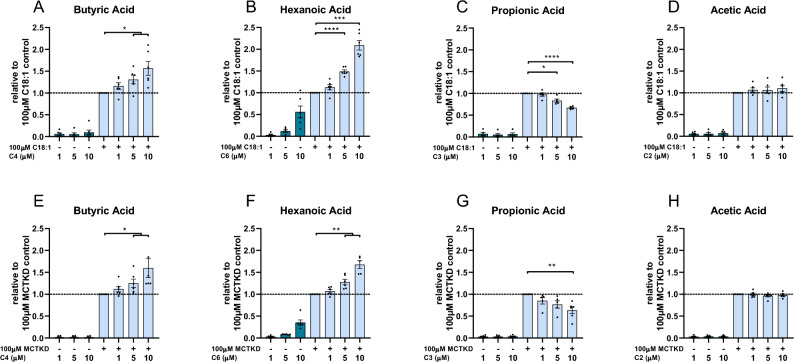
Ketogenic enhancement by butyric acid (C4) and hexanoic acid (C6) and ketogenic restriction by propionic acid (C3). C4, C6, C3, C2 were assessed alone or in combination with either 100 µM C18:1 (**A**–**D**) or 100 µM 6% MCT-KD fatty acid blend (MCTKD) (**E**–**H**) during the ketogenic phase. The βHB yields are normalized to the βHB yield from exposure to 100 µM C18:1 alone (**A**–**D**) or 100 µM MCTKD alone (**E**–**H**) and represent the means ± SEM. The nutrient combinations were compared to incubation in 100 µM C18:1 alone (**A**–**D**) or 100 µM MCTKD alone (**E**–**H**) by one-sample *t* test (*p < 0.05, **p < 0.01, ***p < 0.001, ****p < 0.0001, N = 5–6 independent experiments).

**Figure 5:**
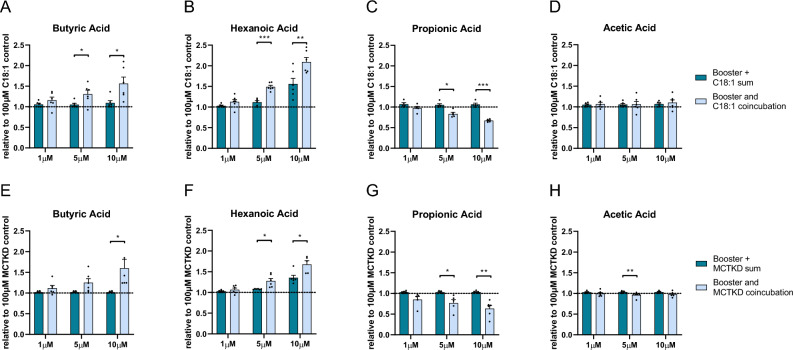
Ketogenic enhancement and restriction effects are more than additive. (**A**–**D**) The βHB yields from incubation with 1 µM, 5 µM and 10 µM butyric acid (C4), hexanoic acid (C6), propionic acid (C3), acetic acid (C2) alone *plus* incubation with 100 µM C18:1 alone are compared to the βHB yields from *co-incubating* the same concentration of C4, C6, C4 or C2 with 100 µM C18:1. The βHB yields are all normalized to the βHB yield from C18:1 alone. (**A**) Co-incubation of 5 µM or 10 µM C4 with 100 µM C18:1 yielded more βHB than the sum of the individual βHB yields from 5 µM or 10 µM C4 and 100 µM C18:1 (p = 0.0406 for 5 µM C4, p = 0.0123 for 10 µM C4, paired *t* test, N = 6 independent experiments). (**B**) Co-incubation of 5 µM or 10 µM C6 with 100 µM C18:1 yielded more βHB than the sum of the individual βHB yields from 5 or 10 µM C6 and 100 µM C18:1 (p = 0.0003 for 5 µM C6, p = 0.0027 for 10 µM C6, paired *t* test; N = 6 independent experiments). (**C**) Co-incubation of 5 µM or 10 µM C3 with 100 µM C18:1 led to a significant lower βHB yield than adding up the individual βHB yields from 5 or 10 µM C3 and 100 µM C18:1 (p = 0.0164 for 5 µM C3, p = 0.0003 for 10 µM C3, paired *t* test; N = 5 independent experiments). (**D**) Co-incubation of 1 µM, 5 µM or 10 µM C2 with 100 µM C18:1 resulted in the same βHB yield than the sum of the individual βHB yields from either 1 µM, 5 µM or 10 µM C2 and 100 µM C18:1 (n.s. by paired *t* test; N = 6 independent experiments). (**E**–**H**) The βHB yields from incubation with 1 µM, 5 µM and 10 µM C2, C3, C4, or C6 alone *plus* incubation with 100 µM 6% MCT-KD fatty acid blend (MCTKD) alone are compared to the βHB yield from *co-incubating* the same concentration of C2, C3, C4 or C6 with 100 µM MCTKD. The βHB yields are all normalized to the βHB yield from MCTKD alone. (**E**) Co-incubation of 10 µM C4 with 100 µM MCTKD yielded more βHB than the sum of the individual βHB yields from 10 µM C4 and 100 µM MCTKD (p = 0.0487 for 10 µM C4, paired *t* test; N = 6 independent experiments). (**F**) Co-incubation of 5 µM or 10 µM C6 with 100 µM MCTKD yielded more βHB than the sum of the individual βHB yields from 5 µM or 10 µM C6 and 100 µM MCTKD (p = 0.0228 for 5 µM C6, p = 0.0159 for 10 µM C6, paired *t* test; N = 6 independent experiments). (**G**) Co-incubation of 5 µM or 10 µM C3 with 100 µM MCTKD resulted in a smaller βHB yield than adding up the individual βHB yields from either 5 µM or 10 µM C3 and 100 µM MCTKD (p = 0.0311 for 5 µM C3, p = 0.0027 for 10 µM C3, paired *t* test; N = 5–6 independent experiments). (**H**) Co-incubation of 5 µM C2 with 100 µM MCTKD modestly decreased the βHB yield compared to the sum of the individual βHB yields from 5 µM C2 and 100 µM MCTKD (p = 0.0089, paired *t* test, N = 5–6 independent experiments). Data is presented as mean ± SEM (*p < 0.05, **p < 0.01, ***p < 0.001).

Next, we evaluated if the assay could detect ketogenic enhancers and inhibitors, defined as compounds that can enhance and inhibit, respectively, the production of ketones from other substrates. To distinguish a regulatory role from a substrate role in ketogenesis, the concentrations of C2, C3, C4 and C6 were applied in a physiological range of 1 to 10 µM, not exceeding reported concentrations in the systemic or portal circulation^27–29^ and when their single substrate assessments showed no or only a mild βHB response (Fig. 2). During the ketogenic phase of the assay, one of C2, C3, C4 or C6 was co-incubated with either 100 µM C18:1 (Fig. 4A–D) or 100 µM 6% MCT-KD (Fig. 4E-H), at which concentration C18:1 (Figs. 1B,C, 2J) or 6% MCT-KD (Fig. 1F) elicited a maximum response. C4 augmented the βHB yield from C18:1 (p = 0.025, 5 µM C4 *plus* 100 µM C18:1 vs 100 µM C18:1; p = 0.016, 10 µM C4 *plus* 100 µM C18:1 vs 100 µM C18:1; one sample *t* test; Fig. 4A) and from 6% MCT-KD (p = 0.047, 5 µM C4 *plus* 100 µM 6% MCT-KD vs 100 µM 6% MCT-KD; p = 0.041, 10 µM C4 *plus* 100 µM 6% MCT-KD vs 100 µM 6% MCT-KD; one sample *t* test; Fig. 4E). C4 alone at 5 µM and 10 µM yielded little (Fig. 4A) to no βHB (Fig. 4E). This suggested that C4 increased the βHB yield from C18:1 and 6% MCT-KD more than additively. To determine whether these co-incubation effects were more than additive, the sum of the individual components was compared to their coincubation, for the co-incubations with 100 µM C18:1 (Fig. 5A-D) or with 100 µM 6% MCT-KD (Fig. 5E-H). The individual βHB yields from C4, C18:1 and 6% MCT-KD were thus compared to the βHB yield from co-incubating C4 with C18:1 or C4 with 6% MCT-KD. Co-incubating C4 with C18:1 (p = 0.041 for 5 µM C4, p = 0.012 for 10 µM C4, paired *t* test; Fig. 5A) or 6% MCT-KD (p = 0.049 for 10 µM C4, paired *t* test; Fig. 5E) increased the βHB yield more than additively. This means that C4 upregulated ketone production from C18:1 and the 6% MCT-KD independently of a use as ketogenic substrate.

A ketogenic enhancer function was also noted for C6. It augmented the βHB yield from C18:1 at 5 µM by 49% (p < 0.0001, 5 µM C6 *plus* 100 µM C18:1 vs 100 µM C18:1, one sample *t* test; Fig. 4B) and at 10 µM by ~ 109% (p = 0.0002, 10 µM C6 *plus* 100 µM C18:1 vs 100 µM C18:1, one sample *t* test; Fig. 4B). Meanwhile, incubation with 5 µM and 10 µM C6 alone yielded ~ 12% and ~ 56%, respectively, of the βHB yield from C18:1, suggesting C6 increased the βHB yield more than additively. Confirming the latter, co-incubation of 5 or 10 µM C6 with C18:1 yielded ~ 33% more βHB than the sum of the individual βHB yields from a matching concentration of C6 and 100 µM C18:1 (p = 0.0003, 5 µM C6; p = 0.0027, 10 µM C6; paired *t* test; Fig. 5B). C6 also augmented the βHB yield from 6% MCT-KD at 5 µM by 28% (p = 0.0062, 5 µM C6 *plus* 100 µM 6% MCT-KD vs 100 µM 6% MCT-KD, one sample *t* test; Fig. 4F) and at 10 µM by ~ 68% (p = 0.0015, 10 µM C6 *plus* 100 µM 6% MCT-KD vs 100 µM 6% MCT-KD, one sample *t* test; Fig. 4F). Meanwhile, incubation with 5 µM and 10 µM C6 alone led to 8% and 35%, respectively, of the βHB yield from 6%-MCT-KD. This suggested C6 increased the βHB yield from 6% MCT-KD more than additively. Supporting this notion, co-incubation of 5 µM or 10 µM C6 with 100 µM 6% MCT-KD yielded 19% and 24% more βHB than the sum of the individual βHB yields from a matching concentration of C6 and 100 µM 6% MCT-KD (p = 0.023, 5 µM C6; p = 0.016, 10 µM C6; paired *t* test; Fig. 5F). These results indicate that C6 increases the βHB yield from C18:1 and the 6% MCT-KD independently of a use as ketogenic substrate.

Propionic acid (C3) decreased dose-dependently the maximum βHB response of C18:1 (p = 0.011, 5 µM C3 *plus* 100 µM C18:1 vs 100 µM C18:1; p < 0.0001, 10 µM C3 *plus* 100 µM C18:1 vs 100 µM C18:1; one-sample *t* test; Fig. 4C). Moreover, the βHB yield from co-incubating C3 with C18:1 was lower than the sum of the individual βHB yields from an equimolar amount of C3 and 100 µM C18:1 (p = 0.016, 5 µM C3; p = 0.0003, 10 µM C3; paired *t* test; Fig. 5C). C3 also decreased dose-dependently the maximum βHB response of 6% MCT-KD (p = 0.052, 5 µM C3 *plus* 100 µM 6% MCT-KD vs 100 µM 6% MCT-KD; p = 0.0049, 10 µM C3 *plus* 100 µM 6% MCT-KD vs 100 µM 6% MCT-KD; one-sample *t* test; Fig. 4G). The βHB yield from co-incubating C3 with 6% MCT-KD was lower than the sum of the individual βHB yields from an equimolar amount of C3 and 100 µM 6% MCT-KD (p = 0.031, 5 µM C3; p = 0.0027, 10 µM C3; paired *t* test; Fig. 5G). These data show that C3 reduced the maximum βHB response of C18:1 and 6% MCT-KD more than subtractively, implying that C3 repressed ketogenesis functionally and not through substrate competition.

C2 did not increase the maximum βHB response of C18:1 or 6% MCT-KD (n.s., one sample *t* test, Fig. 4D,H). Furthermore, the βHB yield from co-incubating C2 with C18:1 was not greater than the sum of the individual βHB yields from an equal concentration of C2 and C18:1 (n.s., paired *t* test; Fig. 5D) and the βHB yield from co-incubating C2 with 6% MCT-KD was similar or even slightly lower than the sum of the individual βHB yields from an equal concentration of C2 and 6% MCT-KD (n.s. for 1 µM C2 and 10 µM C2; p = 0.0089 for 5 µM C2 with a 5.2% reduction for the co-incubation of C2 with 6% MCT-KD compared to the sum of C2 and 6% MCT-KD; paired *t* test; Fig. 5H).

## Discussion

We present a novel hepatocyte ketogenic assay and for the first time a systematic comparison of the ketone yields from individual short, medium, long and very long chain saturated and unsaturated fatty acids and their blends contained in KDs. The assay showed that the ketone yield from individual fatty acids increased with decreasing chain length and peaked at C4. Consistent with this rule, fatty acid blends containing a greater mol% of MCFAs were more ketogenic. The assay identified C6 as a novel ketogenic enhancer and confirmed the ketogenic enhancer activity of C4 and ketogenesis inhibition by C3 described previously, albeit now also at a low physiological concentration. The assay dissociated a dual role as ketogenic enhancer at low concentration from a use as ketogenic substrate at higher concentration.

### Validity of the assay and possible physiological implications

The validity of the assay was demonstrated by (i) establishing concentration–response relationships between ketogenic substrates and βHB responses; (ii) detecting greater maximum responses from single MCFAs than from equimolar amounts of single LCFAs; (iii) increasing the ketone yield from a fatty acid blend by exchanging LCFAs for an equal mol% of MCFAs; (iv) confirming the previously known ketogenic enhancer activity of C4.

The CRCs showed a good fit with low baseline βHB production, indicating there was little internal lipid store utilization for ketone production. All single fatty acid CRCs showed similar EC50s except C4, whose EC50 was ~ twofold higher than the mean. Meanwhile, the CRCs differed clearly in the maximum response. The applicability of this parameter for the selection of fatty acids for optimal ketogenesis depends on whether saturation of ketogenesis from individual fatty acids is reached when KDs are used in patients. For most individual fatty acids tested in the assay, the maximum response is reached at around 100 μM supplementation. Comparing this to plasma levels, daily supplementation with 20 ml MCT oil can increase the C8, C10 and C12 plasma concentrations above 100 µM in healthy humans^30^. Furthermore, in epilepsy patients supplemented with MCT oil at 40 kcal% of their daily energy intake, the mean C8 and C10 plasma concentrations were ~ 280 µM and ~ 150 µM, respectively^31^. However, one should be careful in ingesting such large quantities of MCT oil, as this can lead to gastrointestinal distress, vomiting and diarrhea^32^. A recent study indicates that infants on a KD show a triglyceride plasma concentration between ~ 800 µM and ~ 1190 µM^33^. The plasma levels in these studies suggests that both MCT oil supplementation and triglyceride concentrations during a KD treatment could saturate the liver ketogenic response, highlighting the importance of the maximum response characteristic of individual fatty acids and fat blends. On the other hand, MCT doses between 25 and 85 g lead to linear increases in βHB concentrations in the blood ranging from 0.5–1.8 mM^34^. It would be of interest to know the MCFA concentrations in the human portal vein achieved by the administration of 25–85 g of MCT, however this is difficult to measure in humans. Furthermore, the level of saturation in human hepatocytes could be significantly higher than in rodent liver cells. The question of how the assay results translate into human ketone production requires further validation of dose–response relationships in human hepatocytes or better, in human subjects.

Nonetheless, the extent to which the liver ketogenic response of humans can be extrapolated from the assay readout is unclear. Uptake and intracellular processing kinetics of fatty acids may well differ. Furthermore, the ketone yield from individual fatty acids was assessed in the absence of other nutrients, possibly agonizing, or antagonizing their maximum response.

### Comparing single fatty acids and complex fatty acid blends

In the individual comparison of fatty acids, an overall trend was noted for an increased maximum response with shortened chain-length. However, C2 was an exception to this observation and showed no CRC. C2 is turned into ketones only at supraphysiological concentrations (≥ 1 mM)^35^ not reached in this assay. A possible reason is the high Km of mitochondrial acetyl-CoA synthetase in the liver and subsequently low conversion rates of C2 into acetyl-CoA^36^, the required precursor for ketone biosynthesis.

C4 was the most ketogenic substrate in the assay followed by C6. Both, C4 and C6 were more ketogenic than C8-C12. This is consistent with a study in healthy humans, where a single high dose of C4 increased ketosis to greater extent than a matching or even higher dose of octanoyl-monoacylglycerol^37^. The ketone yields from C8, C10 and C12 were similar in the assay. This contrasts with a study in healthy humans where C8 supplementation was acutely (within 8 h of administration) three and six times more ketogenic than supplementation with equal amounts (20 ml) of C10 and C12, respectively^30^. Furthermore, C8 was more ketogenic than supplementation with equal amounts (20 ml) of MCT oil and coconut oil containing less C8 and more C10 and C12^38^. This mismatch could reflect differences in bioaccessibility in the gut lumen, pre-systemic metabolism in gut cells and/or transport in the portal vein to the liver rather than the uptake and ketogenic processing of C8–C12 by liver cells. The ketone yield declined further for C14–C18. Among the LCFAs, C14 showed the highest maximum response whereas C18, C18:1 and C18:2 had the lowest maximum responses, and C16 appeared between C14 and C18. While saturated fatty acids seem to be preferred substrates for ketogenesis over unsaturated fatty acids in vivo^5^, the degree of saturation did not seem to play a part at the hepatocyte level because C18, C18:1, C18:2 and C18:3 showed similar maximum responses and EC50 values. The inverse relationship between a fatty acid’s carbon chain length and maximum response seems counter-intuitive since a longer carbon chain should provide more acetyl-CoA molecules and ketone bodies produced per fatty acid unit. However, only a fraction of the acetyl-CoA molecules is used for ketone synthesis. VLCFAs require chain-shortening in peroxisomes (peroxisomal β-oxidation), often to C16, before they can cross the inner mitochondrial membrane. This releases one acetyl-CoA molecule through a cycle of four enzymatic reactions. Since acetyl-CoA cannot directly cross the mitochondrial membranes^39^, it may be lost for ketone synthesis. Although LCFAs and even MCFAs may be shortened in peroxisomes^40^, they are less dependent on it for mitochondrial uptake. Secondly, fatty acid transport may impact availability for ketogenesis. LCFAs depend on fatty acid transporters CD36 and FATP5 to cross the cell membrane^41^, and on fatty acid binding proteins for intracellular transport^41^. Furthermore, LCFAs also depend on the rate-limiting carnitine palmitoyltransferase (CPT) system for uptake into the mitochondrial matrix^42^. SCFAs and MCFAs enter the mitochondria at higher rates as they more easily diffuse across the inner mitochondrial membrane and depend less on the CPT system for mitochondrial import^43^. All the above results in faster and greater conversion of SCFA and MCFAs into ketones, and that the fatty acids with the highest carbon chain length do not necessarily have the highest ketogenic potential.

The contribution of C18 and its derivatives in a fat blend to ketogenesis is expectedly low based on their assessment as single fatty acids. They could compete with more effective LCFAs such as C14 or C16. Reducing their relative contribution to the diet may help to optimize ketogenesis. However, this is not straightforward because the raw oils comprise a profile of many different fatty acids and C18 or C18:1 are often over-represented. Furthermore, C18:2 is an essential omega-6 fatty acid in humans and fulfills important physiological roles. Therefore, eliminating C18:2 from the diet is not advisable. Likewise, the essential omega-3 fatty acids C18:3, C20:5 and C22:6 were weak or no ketogenic substrates in the assay, but they are considered essential nutrients in humans.

### Ketogenic enhancement by C4 and C6

Although it is generally known that MCFAs can drive β-oxidation and increase ketogenesis including from LCFAs in perfused fat liver and in vivo^15–17^, our assay unveiled a dual role specifically for C4 and C6 as ketogenic substrates at high concentrations, and ketogenic enhancers at low concentrations. Previous in vitro studies highlighted the effect of C4 on ketone production only at very high concentrations, for example > 800 µM in HepG2 cells^18^. At these levels, it is difficult to distinguish the use of C4 as a substrate or as an enhancer of ketogenesis, which was possible in this new ketogenic assay.

C4 stimulates the expression of FGF21 in HepG2 cells, which in turn drives β-oxidation and ketogenesis in liver^24,25^. C4 inhibits histone deacetylase HDAC3 thereby increasing histone acetylation and transcription of FGF21 by PPARα. However, C6 supplementation failed to up-regulate FGF21^25^, suggesting a different mode of action for C6. In addition to regulating FGF21, C4 could also stimulate the AMPK pathway ^44^ and upregulate Sirtuin 3 (SIRT3) and 5 (SIRT5) albeit such effects would need to be shown in hepatocytes at physiological concentration^45^.

In the context of high-fat diet-induced obesity, C2 was found to stimulate β-oxidation and inhibit lipogenesis in mouse and rat livers^44,46,47^. Since the present assay did not detect a robust enhancement by C2 of the ketone yield from C18:1 or a fatty acid blend, it may require further optimization. For example, induction of β-oxidation genes by C2 could depend on certain fasting- or carbohydrate restriction-induced serum factors (e.g., glucagon), not included in the assay.

### Ketogenic restriction by C3

In the assay, C3 inhibited ketogenesis from C18:1 and a fat blend. Other studies found similar inhibition of ketogenesis from different fatty acid substrates in vitro and in vivo^15,48,49^, however, at supraphysiological C3 concentrations between 2 and 15 mM. In contrast to these studies, the assay detected inhibition by C3 at low physiological concentrations of 5 µM and 10 µM. The ketogenic inhibition could be due to CoA-trapping, whereby propionyl-CoA metabolites are formed and accumulate in the mitochondria^48,50^. The CoA needed to form acyl-CoA for fatty acid oxidation and acetyl-CoA for ketogenesis is depleted, thereby inhibiting ketogenesis. An additional proposed mechanism is the inhibition of β-hydroxy-β-methylglutaryl-CoA synthase, required for ketogenesis^49^. Alternatively, C3 could inhibit ketogenesis in vivo by driving gluconeogenesis in the liver and raising systemic insulin levels^51^. Whether these mechanisms also apply at low concentrations of C3 remains to be clarified.

### Dietary supplementation of C4 and C6

A dual role as enhancers and substrates of ketone synthesis renders C4 and C6 interesting ingredients for ketogenic foods. However, their strong and unpleasant taste and smell challenge their use in nutrition. Furthermore, they may introduce tolerability issues with increased dosing. Taste and smell adversity might be mitigated by encapsulation, for example as tablets or capsules. Another option is the use of C4 and C6 derivatives, for example ketogenic esters of C6, such as Bis-Hexanoyl (R)-1,3-Butanediol that are metabolized to C6 in the gut and increase circulating ketone levels in healthy adults^52^. Another example is tributyrin, composed of three C4 tails bound to a glycerol backbone.

Another route of delivery by KDs could be through dietary fiber fermentation by the gut microbiome. This could be relevant, as one study observed a 55% reduction in total fecal SCFA levels and 20% reduction in C4 levels in epilepsy patients after one month of KD treatment^53^. While a large fraction of the produced SCFAs is locally utilized by colonocytes^54^, a considerable fraction is transported to the liver through the portal vein. One postmortem study shows that the total SCFA concentration in the portal vein is around 300 µM, 148 µM in the hepatic vein and 79 µM in peripheral blood^27^. The contribution of C4 is relatively low, ranging between 14 and 64 µM in the portal vein, 2–32 µM in the hepatic vein and 1–12 µM in peripheral blood^27^. C3 levels range between 17 and 194 µM in the portal vein, 2–69 µM in the hepatic vein and 1–13 µM in peripheral blood^27,55^. Another study shows that portal vein concentrations of single SCFAs rapidly increase after ingestion of 10 g non-absorbable but fermentable sugar lactulose, with on average 39 µM C3 and 27 µM C4 at peak levels^28^. Certain microbiota strains have been reported to produce C6, but to a lesser extent compared to C4^56^. While it is difficult to directly compare in vitro to in vivo concentrations, the C4 levels and C6 levels needed to achieve a ketogenesis-enhancing effect in this assay are very low (5-10 μM). Additionally, C4 could be utilized as ketogenic substrate at these levels, especially during peak fermentation by the microbiome. The aforementioned lactulose is rapidly fermented with peak portal concentrations of C4 already observed after around 30 min^28^, while a more consistent ketogenesis boosting effect may be preferred, and achieved through the selection of dietary fibers that release C4 more slowly.

Dietary fiber supplementation may also contribute to production of C3 by the microbiome, which could counteract the ketogenesis enhancing effect of C4 and C6. Therefore, the selection of dietary fiber mixes may be guided by favorable SCFA production profiles.

The in vitro assay shows promise as a tool to optimize the ketogenic yield of a fat blend. Hence, it could be used to adjust the lipid composition of a ketogenic diet or ketogenic supplement to boost ketosis more effectively. These lipid adjustments could help to reduce the amount of fat ingested in a ketogenic diet while preserving ketosis levels and improve patient compliance with the diet. Additionally, the assay could allow to assess the influence of uneven lipids (e.g. C7 from triheptanoin), drugs, amino acids, glucose and insulin on ketone production. However, the value of the assay to predict liver ketone production and ketosis induction in humans requires clinical validation.

To conclude, the new ketone production assay shows that C4 and C6 are the most ketogenic fatty acids, followed by C8 and C10 in mouse hepatocytes. C4 and C6 acted both as substrate and enhancers of the production of ketones from complex fat blends typically contained in KDs while C3 blocked ketone production. The supplementation of a KD with C4 or C6 therefore promises to enhance ketosis induction in humans. Since the intake of C4 or C6 is potentially associated with sensory or gastrointestinal discomfort, their production could be induced in humans endogenously by the gut microbiota through the intake of fermentable fibers or fiber blends. Human trials are however needed to validate the findings made in the current assay and advise on the intake and dosing of specific fatty acid or fiber type blends to optimize ketone production in humans.

## Data Availability

The datasets used and/or analysed during the current study are available from the corresponding author on reasonable request.

## Abbreviations

CPT: Carnitine palmitoyltransferase
cKD: Classic ketogenic diet
CRC: Concentration response curve
DPBS: Dulbecco’s phosphate-buffered saline
DRE: Drug-resistant epilepsy
LGIT: Low-glycemic index treatment
β-oxidation: Fatty acid oxidation
GI: Glycemic index
βHB: β-Hydroxybutyrate
KHB: Krebs Henseleit Buffer
LCFAs: Long chain fatty acids
MCFAs: Medium chain fatty acids
MCT-KD: Medium chain triglyceride ketogenic diet
MCTs: Medium chain triglycerides
MAD: Modified Atkins Diet
Pen-Strep: Penicillin-Streptomycin
SCFAs: Short-chain fatty acids
